# Estimating Causal Effects from Family Planning Health Communication Campaigns Using Panel Data: The “Your Health, Your Wealth” Campaign in Egypt

**DOI:** 10.1371/journal.pone.0046138

**Published:** 2012-09-25

**Authors:** Paul L. Hutchinson, Dominique Meekers

**Affiliations:** Department of Global Health Systems and Development, School of Public Health and Tropical Medicine, Tulane University, New Orleans, Louisiana, United States of America; Tulane University School of Public Health and Tropical Medicine, United States of America

## Abstract

**Background:**

Health communication campaigns – involving mass media and interpersonal communication - have long been utilized by national family planning programs to create awareness about contraceptive methods, to shift social norms related to fertility control, and to promote specific behaviors, such as the use of condoms, injectable methods or permanent sterilization. However, demonstrating the effectiveness of these campaigns is often complicated because the infeasibility of experimental designs generally yields statistically non-equivalent samples of campaign-exposed and unexposed individuals.

**Methods:**

Using data from a panel survey of reproductive age women in Egypt, we estimate the effects of the multimedia health communication campaign “Your Health, Your Wealth” (“Sahatek Sarwetek”) on precursors to contraceptive use (e.g., spousal communication, birth spacing attitudes) and on modern contraceptive use. Difference-in-differences and fixed effects estimators that exploit the panel nature of the data are employed to control for both observed and unobserved heterogeneity in the sample of women who self-report recall of the messages, thereby potentially improving upon methods that make no such controls or that rely solely on cross-sectional data.

**Findings:**

All of the estimators find positive effects of the “Your Health, Your Wealth” campaign on reproductive health outcomes, though the magnitudes of those effects diverge, often considerably. Difference-in-differences estimators find that exposure to the campaign increases the likelihood of spousal discussions by 14.4 percentage points (pp.) (SE = .039, p<0.001) but has no effect on contraceptive use. In contrast, the fixed effects, instrumental variables estimator, controlling for unobserved heterogeneity, finds a large, statistically significant effect on modern contraceptive use (27.4 pp., SE = 0.135, p = 0.043).

**Conclusions:**

The difficulties of evaluating family planning communication programs may be surmountable using panel data and analytic methods that address both observed and unobserved heterogeneity in exposure. Not controlling for such effects may lead to substantial underestimates of the effectiveness of such campaigns.

## Introduction

Health communication campaigns have long been integral components of national family planning programs. Mass media, counseling and other forms of interpersonal communication have been widely used to inform and create awareness about family planning methods and their availability, to entertain populations and establish influential role models, and to promote specific behaviors, such as the use of condoms, injectable methods or permanent sterilization [1]–[9]. However, evaluations of these programs have frequently been plagued by a number of difficulties, which we address by using data from a panel of reproductive age women in Egypt to estimate the effects of the “Your Health, Your Wealth” national multimedia campaign on contraceptive use and attitudes.

At the heart of the problem for the evaluation of many large-scale health communication interventions is the inability or impracticality of using experimental designs in which individuals are randomized into exposed treatment groups and unexposed control groups. The use of randomization and experimental research designs is the predominant mechanism for inferring causal relationships because they enable a set of control individuals – equivalent in all respects except exposure to an intervention – to represent the counterfactual outcome for treatment individuals had they not received the intervention. Causal impacts are therefore measured as the difference in mean outcomes between treatment and control individuals [10], [11]. But randomization is rarely employed in the evaluation of communication interventions because those interventions often cover entire countries, potentially exposing all targeted individuals, or because localized interventions risk contamination across geographic areas, or because ethical concerns proscribe limiting dissemination of health messages to a subset of potential beneficiaries [11]–[16].

In the absence of randomized control designs, evaluations of health communication programs have frequently adopted alternative methods to generate inferences surrounding causal relationships, generally using non-equivalent comparison groups and statistical methods that seek to achieve equivalence based on observed characteristics of exposed and unexposed individuals. In many cases, comparison groups can be generated because health communication programs – even those that attempt to target all members of a population - are likely to leave some sub-population unexposed to the intervention, as some individuals may be less regular consumers of media than others or may not recall having been exposed. Population surveys, such as the Demographic Health Surveys or more focused communication surveys, can be used to identify individuals who recall being exposed to campaign messages and individuals who do not, while also collecting information on their health behaviors and outcomes. A common choice for a measure of a communication intervention’s effect involves a comparison of average outcomes for those who recall being exposed to intervention messages relative to those who do not. This is the approach taken by numerous evaluations of health communication programs, which use single equation multivariate regression models with cross sectional data to measure the effect of exposure to a health communication program on a family planning outcome of interest, controlling for a limited set of observable characteristics of those individuals [17]–[23].

Such a measure, however, may contain very serious limitations, as the sample of unexposed individuals may be very different than the sample of exposed in ways that may also affect outcomes under study. As noted in other studies [15], [24], [25], exposed individuals likely differ from unexposed individuals in measurable (exogenous) ways, such as levels of education, income, age, or geographic location. But they may also differ in other less easily measured ways. For example, previous researchers have argued that family planning programs are often targeted to specific populations such as those that are hard-to-reach, those with higher fertility or those that have more traditional norms regarding family planning use. Such targeting – to the extent that researchers cannot quantify or control for it – may bias estimates of campaign effectiveness if the targeting is also related to program outcomes [26]–[29]. Alternatively, individuals exposed to program messages may be more media savvy, or they may simply have higher levels of motivation to control their fertility. As a result, they may represent a form of low-hanging fruit - audiences that are more receptive to family planning communication messages, implying that attempts to replicate program successes with alternative populations may be less effective. In short, the sample of individuals who self-report exposure to family planning messages may be very different from the sample of unexposed individuals in ways that may bias estimates of campaign effectiveness.

Other researchers have attempted to overcome the limitations of regression models [25], [30] using matching methods that draw a cohort of non-intervention individuals from some population – either the same population or a similar enough comparison group – who can be “matched” with intervention individuals of similar characteristics. Average outcomes for untreated matched groups serve as the relevant counterfactual to the missing outcomes of exposed program participants. Program effects, as in an experiment, are measured as the difference in average outcomes for these two groups. Nonetheless, while intuitively appealing, matching methods and multivariate regression models share the same conditional independence assumption - that selection into the treatment group is determined by observable characteristics while unobserved characteristics that may affect family planning outcomes are distributed randomly across exposed and unexposed individuals [31], [32]. Hence, they are likely to face similar biases.

The fundamental difficulty, in short, is that evaluations of health communication programs often rely upon measures of program exposure that are at least in part determined by the actions, choices and characteristics of the potential beneficiaries, which may therefore confound estimates of the health communication intervention’s effectiveness. In such cases, naïve estimators that assume exogenous exposure – or exogeneity conditional on a limited set of control variables - may be severely biased.

To address non-random program exposure, researchers have several alternatives, which have seldom been used in the evaluation of the family planning communication literature but which we explore here. These involve, for example, the use of instrumental variables approaches to create exogenous variation in exposure by identifying a set of variables that affect exposure to the intervention but not the outcome itself, thereby purging estimates of the program effect from the confounding effects of the determinants of exposure [15], [33], [34]. In previous analyses, such instruments have included measures of frequency of exposure to different media [24], [30]. Alternatively, fixed effects models with a panel of exposed and unexposed individuals allow researchers to collect information on potential program beneficiaries at a baseline, again at a post-intervention time point, and then to compare changes in outcomes for those individuals who were exposed to the intervention relative to those who were not exposed. Under the assumption that unobserved heterogeneity affecting program exposure is time invariant, evaluators can better attribute changes in family planning outcomes to the program rather than to confounding by observed or unobserved characteristics of respondents [35], [36].

In this paper, we make use of panel data to estimate the effects of the “Your Health, Your Wealth” (*Sahatek, Sarwetek*) health communication program in Egypt while controlling for unobserved heterogeneity between exposed and unexposed respondents. The “Your Health, Your Wealth” campaign was a component of the Communication for Healthy Living (CHL) project in Egypt, reflecting the cooperation of the Egyptian Ministry of Health and the the United States Agency for International Development (USAID). CHL in turn was one part of the Health Communication Partnership (HCP), a global health communication initiative funded by USAID. The CHL program supported activities at both the national and local level in the areas of family planning and reproductive health, maternal and child health, infectious diseases control, healthy lifestyle, household preventive health, and health maintenance practices. Messages have been disseminated via a national integrated health communication campaign using television, radio, press advertising and public affairs programming. The “Your Health, Your Wealth” campaign involved national multimedia and community-based interventions aimed at encouraging families to engage in healthy behaviors at different points in the life stage. A key component of the overall communication strategy was the “Mabrouk!” (Congratulations!) Initiative, targeted towards newlyweds as a strategic entry point for encouraging behaviors that promote healthy families, including important events such as pregnancy, labor and delivery, postpartum infant care, family planning, and overall family health [37], [38]. Specific family planning messages include the benefits of birth spacing and the need for post-partum resumption or initiation of family planning to avoid early pregnancy [39], [40].

We focus here on estimating the effects of the “Your Health, Your Wealth” (Sahatek, Sarwetek) health communication campaign on several family planning outcomes, including current contraceptive use, discussions with a spouse about family planning and the use of family planning for birth spacing, and agreement with statements about the benefits of family planning for birth spacing. Several estimation methods are used to attempt to develop and to compare estimates of the causal effect of exposure to the “Your Health, Your Wealth” national multimedia health communication campaign: (1) a single-equation cross sectional estimator (using endline data), (2) matching on the propensity score of exposure, (3) a difference-in-differences estimator, (4) a fixed effects estimator, (5) a fixed effects, instrumental variables estimator, and (6) a bivariate probit model that models simultaneously both the outcome and exposure equations and allows for correlation in unobservables across the two equations. We find that all estimators – including those relying solely on the post-only cross section and those assuming exogenous campaign exposure – yield positive effects of campaign exposure, though the effects diverge in magnitude and statistical significance. Statistical tests indicate that endogenous exposure may in fact be problematic, in which case the naïve estimators assuming exogeneity may underestimate actual campaign effects.

## Data

### Data Source

We use data from two waves of the Menya Village Health Surveys conducted in seven villages of Menya Governorate in Egypt in 2004 and 2005. All villages were exposed to the national “Your Health, Your Wealth” multimedia health communication campaign, which was broad on national television. However, five of these villages received more intensive community-based interventions from CHL, while two villages were used as comparison villages. The intensive community-based interventions were implemented through community development associations (CDAs), and included activities such as newlywed visits, pregnancy classes, safe delivery referrals, and postpartum home visits [Hess, Meekers, Storey 2012]. The home visits provided an opportunity to reinforce national media messages through interpersonal communication, and to encourage women to start using family planning within forty days after the delivery and to space their children three years apart. The surveys were funded by the United States Agency for International Development (USAID) as part of the external evaluation of the impact of the Health Communication Partnership (HCP). This evaluation – part of a multi-country study – was conducted by Tulane University’s Department for International Health and Development (Tulane/IHD), School of Public Health and Tropical Medicine. Collection of data was undertaken by El-Zanaty and Associates.

Our analysis focuses on ever-married women aged 15–49 years. For the 2005 sample, women who were interviewed in 2004 and completed 50 years by the date of the interview in 2005 were excluded. Only usual household residents were eligible for interview.

Two types of questionnaires were used in the data collection: (1) a household questionnaire which identified eligible respondents and collected information on household socioeconomic characteristics and living conditions and (2) eligible respondent questionnaires which focused on health knowledge, attitudes and behaviors, as well as detailed questions about exposure to different health communication messages and campaigns.

A multi-stage cluster sample design was used to identify respondents. At the first stage, five intervention villages receiving more intensive community support (Zohra, Saft El khamar El sharkia, Nazlet Hussein Ali, Monshaat El Maghalka, and Koloba) and two control villages (Toukh El khail and Ebshedat) were selected. At the second stage, each village was divided into segments of approximately 1000 households. Each village had 10 segments, except for Koloba (which had 11 segments) and Ebshedat (which had 13). One segment was then selected at random, and a household listing was conducted by El-Zanaty and Associates. At the third stage, approximately 35 households were systematically sampled at random from the household listing. The sampling interval was determined by dividing the total number of households in each segment by 35 [39], [40].

#### Ethics statement

Verbal consent was obtained from all survey respondents. Verbal – rather than written - consent was obtained because of the low literacy levels of many of the respondents. Prior to commencing an interview, survey interviewers read a consent statement to potential respondents. The consent statement explained the reasons for the survey and the rights of respondents, informed respondents of the estimated length of time for an interview, described procedures to maintain confidentiality, and listed contact information for survey administrators if respondents had subsequent questions or concerns. Interviewers then documented with a check mark and a signature on the questionnaire to indicate whether or not consent had been given by a respondent. This consent process, as well as the study design, research protocol, and questionnaires were reviewed and approved by the Tulane University Biomedical Institutional Review Board (IRB) prior to the implementation of fieldwork in order to ensure that they met the qualifications and restrictions of the Tulane University Human Research Subject Protection Program.

Fieldwork for the 2004 MVHS was conducted over three weeks beginning in late July and ending in mid-August 2004. The 2005 MVHS was conducted over a two-week period in August and September 2005. For quality control, 5 percent of the sample was selected for re-interview using shorter versions of the original questionnaires. The re-interviews occurred following the main fieldwork and involved special teams that did not involve the original interviewers. During the re-interviews, teams also attempted to visit households or individuals whose interviews were not completed during the initial village visits.

Attrition across the two waves was negligible. In 2004, 2,316 households were selected for interview, and 2,298 households were interviewed. In 2005, 205 of the original households were no longer eligible, while 2,093 of the 2004 households were re-interviewed and 126 new households were added to the sample. A total of 2,240 ever-married women were interviewed in 2004 (response rate of 99.7 percent). By 2005, 2,073 of these women were still eligible, while an additional 86 youth were married and became eligible for interview and 201 women had a new husband and were also eligible. Of these women, a total of 2,284 were successfully interviewed (response rate of 96.8 percent).

## Methodology

In this analysis, we focus on several outcomes related to family planning and use of family planning in Egypt. Specifically, we examine whether or not a woman is currently using modern family planning (i.e., oral contraceptives, IUD, injectables, implants, or diaphragm), whether or not she had a discussion with her spouse about family planning in the past 12 months, whether or not she has discussed the use of family planning for birth spacing, and whether or not she agrees with statements about the benefits of family planning for birth spacing. These outcomes are chosen because they reflect specific message themes from the “Your Health, Your Wealth” campaign.

As our measure of exposure to the “Your Health, Your Wealth” campaign, we use the variable from the 2005 wave of the survey indicating whether or not the respondent reported having seen “Your Health, Your Wealth” messages in the last 12 months and specifically mentioned that those messages pertained to either “birth spacing” or “family planning use in the 40 days following birth.” In our sample of 2,088 women in 2005, 378 (18.1%) recalled having seen either messages; 321 (15.4%) recalled the messages related to postpartum family planning use, and 151 (7.2%) recalled the messages related to birth spacing.

### Estimation Assuming Exogenous Exposure

As a starting point, we focus on estimation methods that assume that exposure to the “Your Health, Your Wealth” campaign is exogenous, that is, once we have controlled for observed characteristics of respondents, there are no other factors that simultaneously determine both exposure to the campaign and our family planning outcomes. We estimate equations for each of the four family planning outcomes using a combined exposed-unexposed group sample and include variables representing self-reported exposure to the “Your Health, Your Wealth” campaign and a set of exogenous controls. We estimate models separately for each of the two cross-sectional waves of the surveys. Using the first wave of data allows us to test for initial differences in the samples of exposed and unexposed respondents prior to the implementation of the communication campaign, while estimation with the second wave provides us with an estimate of the association between exposure and family planning outcomes after the campaign had been in operation for 18 months.
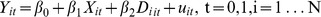
(1)


In this specification, Y_it_ is the outcome of interest (e.g., current use of modern family planning) for i = 1…N individuals in the sample at time t = 0 (2004) or t = 1 (2005). The X_it_ represent a vector of exogenous control variables (e.g., wealth, education, village characteristics), D_it_ represents self-reported exposure to the “Your Health, Your Wealth” program, and u_it_ is a measure of unobservables associated with the outcome Y_it_ and assumed in this specification to be uncorrelated with the program exposure variable D_it_. The parameter β_2_ represents a measure of the cross-sectional association of program exposure with the outcome Y_it_, controlling for the exogenous control variables X_it_.

Each of the outcome variables is binary, i.e., Y_it_ = 1 if the individual engages in the behavior (e.g. is currently using modern family planning) and Y_it_ = 0 otherwise. We make a similar assumption about exposure to the health communication program: a woman either recalls hearing or seeing the “Your Health, Your Wealth” messages (D_it_ = 1) or not (D_it_ = 0). We model the response probability Pr[Y_it_ = 1|X_it_, D_it_] as a logit model.

Key explanatory variables in these multivariate models include a categorical variable for a woman’s level of education (none, primary or secondary/university), a categorical variable for a woman’s age (in 5- or 10-year increments), a categorical variable of household wealth constructed from a principal components analysis of household ownership of a set of consumer durables, a continuous variable for the number of children ever born to a woman, the presence in the village of a recognizable leader, and residence in a program village (relative to a non-program village).

This approach has two key limitations. First, it assumes that exposure to “Your Health, Your Wealth” messages is exogenous once socio-demographic control variables are included in the model. However, as previously mentioned, exposed respondents may differ from unexposed respondents in many important unmeasured ways – differing fertility experiences, differing norms related to family planning, or differing motivations to control their fertility (e.g., gender preferences for children). Such unmeasured factors can influence both campaign exposure and family planning, leading to estimates of β_2_ that are seriously under- or over-estimated. Further, the single equation cross-sectional model is unable to assess how exposure to the campaign is associated with changes across time in the family planning indicators.

#### Differences-in-differences (DID) estimator

In contrast to the previous model, the difference-in-differences estimator uses the full panel sample of baseline and endline observations to estimate the effect of the campaign for intervention “exposed” individuals relative to comparison “unexposed” individuals. The measure of the causal effect is represented by the coefficient on the interaction term β_4_ in a regression of the family planning outcome on a year dummy variable T_t_, self-reported exposure to the “Your Health, Your Wealth” program D_it_, their interaction 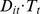, and a set of controls.
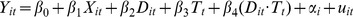
(2)


As with the cross sectional model, we again model the response probability Pr[Y_it_ = 1|X_it_, D_it_, T_t_] as a logit.

While the DID estimator has the advantage that it addresses how exposure to the program may be associated with changes in family planning outcomes, it is unable to confront possible correlation between unobserved factors (e.g., motivations, family planning experience) and family planning outcomes that may bias estimates of the campaign’s effects.

#### Propensity score matching (PSM)

A method with a similar motivation involves matching individuals with similar likelihoods of exposure to the campaign at baseline and then comparing average outcomes at endline. Matching methods reduce bias from non-random treatment assignment by balancing on observed covariates [31], [41]–[43]. A central assumption of matching methods is that treatment assignment is strongly ignorable, i.e., that assignment and outcomes are independent conditional upon measured characteristics of survey respondents [44], an assumption shared by multivariate regression models [32].

The propensity score is estimated as a function of a set of pre-determined (baseline) characteristics of respondents hypothesized to be independent of the ultimate outcomes: age, education, wealth, presence of a recognizable village leader, being in a program village. In this analysis, the propensity score is constructed and tests of covariate balance are performed using the STATA 12.0 command *pscore* [41]. We estimate the average treatment on the treated (ATT) effect using kernel matching with the STATA 12.0 command *psmatch2* [45]. The kernel matching procedure uses a weighted average of all controls, where the weights are inversely proportional to the distance between the propensity score of treated individuals and control individuals [41]. We restrict our matching to the area of common support between exposed and unexposed respondents. Overall, all 378 exposed respondents were matched.

### Estimation Controlling for Endogenous Exposure

In addition to the above methods assuming exogenous program exposure, we also use the following estimators that assume and test for endogenous exposure:

#### Fixed effects logit

As with the DID and PSM estimators, we make use of the panel nature of the data to estimate a fixed effects logit model using conditional maximum likelihood. In other words, information at the 2005 endline for our sample of 2,088 women is linked with their 2004 baseline information. In the fixed effects model, the error term in equation (1) can be expanded to include both a time-invariant individual-specific effect α_i_ and time-varying component u_it._ A time period specific effect T_t_ is also included as in the DID model.
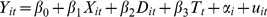
(3)


In the fixed effects model, the correlation between the exposure variable D_it_ and the error term is assumed to be with the time invariant component α_i_. In other words, exposure may be higher among people with certain types of fertility experiences, motivations to use family planning, or norms surrounding the use of family planning but such relationships are constant across time. In this framework, parameters are estimated using the conditional likelihood function constructed from observations in which Y_it_ varies from time period 1 to 2. For these observations, the individual effects α_i_ can be shown to drop out of the probability density in the likelihood function, thereby removing possible confounding by time invariant motivations, experiences and norms and yielding a consistent estimate of the conditional maximum likelihood estimator of β_2_. An important drawback of this estimation method, however, is that the effects of variables that do not vary across time cannot be determined [46], [47]. Further, bias in parameter estimates may still result if correlation between exposure and the unobservables rests with the time varying error term 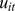.

#### Fixed effects, instrumental variables panel data model

This model extends the previous fixed effects model to include the possibility of endogenous regressors. In the previous model, the correlation between exposure and the unobservables is assumed to be with the time invariant unobservable term *α_i_*.representing attitudes, motivations, or experiences that are fixed across time. This is a potentially problematic assumption as fertility experiences may alter a woman’s motivations or attitudes towards family planning in ways that may also be associated with exposure to the campaign. As a consequence, we employ an instrumental variable approach, which involves estimating an equation for program exposure:
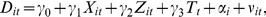
(4)where the X_it_ represent an overlapping vector of exogenous control variables that also affect the outcome Y_it_, the Z_it_ are a non-overlapping vector of variables that are correlated with D_it_ but not Y_it_, and v_it_ is a measure of unobservables associated with D_it_ but also potentially correlated with u_it_ in equation (1). Because of this correlation, estimation of the parameter β_2_ in equation (3) – as well as the other parameters of the model - may be biased by some measure of the degree of correlation in the unobservables affecting both D_it_ and Y_it_. Estimation of equation (4) is then conducted simultaneously with the family planning outcome equation (3) via the method of Limited Information Maximum Likelihood (LIML) [48]. In theory, instrumental variables estimators can provide consistent estimates of program effects, assuming that valid exclusion restrictions for the instrumental variables can be identified. In reality, however, identifying suitable instruments is not without some degree of difficulty [15], [33], [34].

To achieve identification in this model, we include variables hypothesized to be statistically associated with exposure to “Your Health, Your Wealth” (D_it_) but not with the outcomes under study (Y_it_). These include variables for whether or not a woman lived in a designated program village, whether or not a woman was willing to participate in community-organized activities to improve family health, and whether or not a woman had ever heard of community gatherings to discuss health and family planning. These variables were chosen as proxies for informal communication about family planning. Estimation involves use of the full panel of treatment and control individuals [44], [49] and is performed in Stata 12.0 using the *xtivreg2* estimator. Model identification was determined by constructing the Kleibergen-Paap rk Wald F statistic [48]. These values are then compared with the Stock-Yogo critical values [50].

#### Bivariate probit

As a final alternative, we estimate both equations (1) and (4) simultaneously using a bivariate probit model for two binary outcomes. The model is motivated using a continuous underlying latent variable specification for both exposure to “Your Health, Your Wealth” and the family planning outcome, whose discrete realizations are given as above by D_it_ and Y_it_ respectively. In equations (1) and (4), the disturbance terms u_i_ and v_i_ are assumed to be joint normal with means of 0 and variances of 1. The likelihood function is constructed as the product of the four mutually exclusive outcomes – (Y_it_ = 1, D_it_ = 1), (Y_it_ = 1, D_it_ = 0), (Y_it_ = 0, D_it_ = 1), and (Y_it_ = 0, D_it_ = 0). Importantly, this specification allows for correlation ρ between the unobservables in the two equations, thereby controlling for unobserved heterogeneity in the samples of self-reported exposed and unexposed individuals. Standard statistical software provide an estimate of ρ as part of standard regression output. We estimate the bivariate probit model using the Stata 12.0 command *cmp* developed by Roodman [51] for conditional recursive mixed-process estimators. We use the Likelihood Ratio test proposed by Buis [52] to test for the exogeneity of exposure.

## Results

### Descriptive Results

Characteristics of the samples of exposed and unexposed women for 2005 are shown in Table 1. On average, women who reported having seen “Your Health, Your Wealth” were approximately 3 years younger (29.76 years versus 32.80 years, p<0.001), had fewer children (3.40 versus 4.09, p<0.001), were more likely to report that there was a leader in their community (24.3% versus 16.5%, p<0.001), and were less likely to live in a treatment village (62.7% versus 70.9%, p = 0.002). Exposed women were more educated; 29.9% had a secondary or higher level of education versus 19.0% of unexposed women. They were also wealthier on average; 25.7% of exposed women were in the highest wealth quintile versus 20.3% of unexposed women.

**Table 1:** Descriptive Statistics.

	Heard YHYW		Didn’t Hear YHYW		
Characteristic	Pct	N	Pct	N	p
Age (years)					
15–19	6.9%	26	4.1%	70	
20–24	24.1%	91	18.4%	315	
25–29	25.1%	95	19.2%	327	
30–34	14.0%	53	16.2%	276	
35–39	15.6%	59	14.9%	254	
40–44	7.7%	29	12.5%	214	
45–49	6.6%	25	14.8%	252	<0.001
Mean Age (years)	29.76		32.80		<0.001
Children Ever Born (mean)	3.40		4.09		<0.001
Wealth Quintile					
Poorest	10.9%	41	21.0%	358	
2nd Poorest	21.0%	79	20.1%	342	
Middle	22.0%	83	19.5%	332	
2nd Wealthiest	20.4%	77	19.1%	326	
Wealthiest	25.7%	97	20.3%	346	<0.001
Education					
None	52.4%	198	60.5%	1,035	
Primary	17.7%	67	20.5%	351	
Secondary or Above	29.9%	113	19.0%	324	<0.001
Community Leader					
No	75.7%	286	83.5%	1,428	
Yes	24.3%	92	16.5%	282	<0.001
Treatment Village					
No	37.3%	141	29.1%	497	
Yes	62.7%	237	70.9%	1,213	0.002
Total		378		1708	

Table 2 presents outcomes for 2004 and 2005 for those who recalled exposure to the “Your Health, Your Wealth” messages (in 2005) as well as for those who did not. At the baseline, few statistically significant differences in family planning outcomes were observed. For example, contraceptive use was nearly identical - 39.2 percent of exposed ever married women versus 40.3 percent of unexposed women. The only statistically significant difference was for the variable “Discussed birth spacing in the last 6 months” –36.8 percent for exposed women versus 31.1 percent for unexposed women (p = .031).

**Table 2:** Family Planning Outcomes for Women, by exposure to the “Your Health, Your Wealth” messages, 2004 and 2005.

	MVHS 2004			MVHS 2005			2004–2005	
	Exposed	Unexposed	p	Exposed	Unexposed	p	Diff	p
Modern Contraceptive Use	0.392	0.403	0.682	0.476	0.442	0.228	0.046	0.088
Discussed FP with partner inlast 6 months	0.386	0.377	0.743	0.386	0.226	<0.001	0.151	<0.001
Discussed birth spacing inlast 6 months	0.368	0.311	0.031	0.349	0.199	<0.001	0.093	0.006
Agree that spacing improveschild health	0.759	0.753	0.805	0.780	0.692	<0.001	0.083	0.023
N		1,708		378	1,708			

At the endline, statistically significant differences were observed for three of the four outcomes. The lone exception was for modern contraceptive use –47.6 percent for the exposed relative to 44.2 percent for the unexposed (p = .228). Nonetheless, the changes across time for the exposed relative to the unexposed – equivalent to the difference-in-differences model without controls - were statistically significant for each of the outcomes. For example, the difference-in-differences estimate of the program effect on modern contraceptive use was 4.5 percentage points (p = 0.148), while the estimates for “Discussed birth spacing in the last 6 months” and “Agrees that spacing improves child health” were 9.3 (p = 0.010) and 8.3 (p = 0.049) percentage points respectively. The largest effect was for “Discussed FP with partner in the last 6 months” which showed a 15 percentage point difference (p<0.001) between treatment and comparison, due entirely to a significant decrease in the prevalence of discussion for the comparison group.

### Estimation Results

Table 3 summarizes the marginal effects for each of the estimation methods. Full regression results for each outcome and estimation method are shown in Tables 4, 5, 6, 7, 8. As noted above, all estimators – with the exception of the 2004 cross sectional estimator - yielded positive effects of campaign exposure, though the effects diverged in magnitude and statistical significance. We show via several statistical tests that endogenous exposure may in fact be problematic, in which case the naïve estimators assuming exogeneity may underestimate actual campaign effects.

**Table 3:** Marginal effects of exposure to “Your Health, Your Wealth,” by estimation method and family planning outcome.

Method		ModernContraceptive Use	Discuss FP with spouse	Discuss birth spacing	Agree that spacing is healthy
***Methods assuming exogenous exposure***					
2004 Cross Section	dy/dx	**−0.001**	**−0.021**	**0.032**	**0.015**
	SE	0.027	0.026	0.026	0.024
	P	0.973	0.434	0.213	0.640
2005 Cross Section	dy/dx	**0.039**	**0.107**	**0.108**	**0.068**
	SE	0.027	0.210	0.021	0.027
	P	0.147	<0.001	<0.001	0.012
Difference in differences – no controls	dy/dx	**0.045**	**0.151**	**0.093**	**0.083**
	SE	0.031	0.038	0.035	0.041
	P	0.148	<0.001	0.010	0.049
Difference in differences with controls	dy/dx	**0.029**	**0.144**	**0.086**	**0.076**
	SE	0.031	0.039	0.037	0.041
	P	0.359	<0.001	0.021	0.068
Propensity Score Matching	ATT	**0.040**	**0.154**	**0.094**	**0.095**
	SE	0.069	0.064	0.061	0.062
	P	0.561	0.017	0.125	0.125
***Methods with controls for endogenous exposure***					
Fixed Effects Logit	dy/dx	**0.011**	**0.106**	**0.149**	**0.045**
	SE	0.009	0.037	0.043	0.047
	P	0.223	0.005	0.001	0.343
Bivariate probit (cmp)	dy/dx	**0.231**	**0.425**	**0.429**	**0.178**
	SE	0.076	0.040	0.042	0.091
	P	0.002	0.000	0.000	0.049
Test for exogeneity: LR chi2(1)		6.09	25.53	24.27	1.84
Prob>chi2		0.014	0.000	0.000	0.175
Fixed Effects IV Estimator	dy/dx	**0.274**	**0.614**	**0.229**	**0.270**
	SE	0.135	0.199	0.301	0.267
	P	0.043	0.002	0.446	0.311
Test for exogeneity: LR chi2(1)		2.956	3.135	0.831	1.192
Prob>chi2		0.086	0.077	0.362	0.275

As a starting point, we ran a single-equation logit estimation of each family planning outcome on the exposure variable and a set of control variables using the pre-intervention 2004 sample only (Table 3). For none of the outcomes was the program exposure variable statistically significant, providing a general indication that – conditional on the controls – there was little baseline evidence of unobserved heterogeneity.

**Table 4:** Cross Sectional Logit Estimates, 2005.

	Modern Contraception			Discuss FP			Discuss Birth Spacing			Spacing is healthy		
	Coef.	Std. Err.	P>z	Coef.	Std. Err.	P>z	Coef.	Std. Err.	P>z	Coef.	Std. Err.	P>z
**Hear YHYW**	**0.178**	**0.123**	**0.147**	**0.654**	**0.131**	**0.000**	**0.673**	**0.131**	**0.000**	**0.354**	**0.142**	**0.013**
(Base: Age = 15–19)Age 20–24	0.305	0.254	0.230	−0.422	0.235	0.073	0.003	0.259	0.991	0.463	0.254	0.068
Age 25–29	0.096	0.258	0.711	−0.658	0.243	0.007	−0.006	0.264	0.980	0.221	0.255	0.386
Age 30–34	0.706	0.272	0.009	−1.033	0.270	0.000	−0.010	0.286	0.973	0.310	0.273	0.256
Age 35–39	0.331	0.284	0.243	−2.011	0.306	0.000	−0.304	0.305	0.319	0.426	0.286	0.136
Age 40–44	−0.199	0.300	0.508	−2.760	0.363	0.000	−0.200	0.325	0.539	0.491	0.303	0.105
Age 45–49	−1.568	0.327	0.000	−4.656	0.555	0.000	−0.606	0.345	0.079	0.126	0.307	0.680
Children Ever Born	0.263	0.027	0.000	0.136	0.035	0.000	−0.051	0.031	0.104	−0.019	0.027	0.466
(Base: Poorest Quint)2^nd^ Poorest Quintile	0.056	0.151	0.714	0.172	0.182	0.345	0.699	0.221	0.002	0.046	0.159	0.771
Middle Quintile	0.095	0.153	0.535	0.242	0.181	0.181	0.827	0.219	0.000	0.160	0.160	0.319
2^nd^ Wealthiest Quintile	0.220	0.155	0.157	0.299	0.184	0.103	1.466	0.212	0.000	0.237	0.162	0.145
Wealthiest Quintile	0.121	0.152	0.428	0.267	0.182	0.143	1.454	0.209	0.000	0.791	0.168	0.000
(Base: No Education)Primary Education	−0.122	0.131	0.349	−0.057	0.162	0.725	0.261	0.149	0.080	0.042	0.138	0.760
Secondary Education	0.001	0.252	0.996	0.005	0.263	0.986	−0.167	0.282	0.553	−0.135	0.272	0.619
Community Leader	0.069	0.123	0.575	0.037	0.142	0.793	0.107	0.141	0.446	−0.225	0.133	0.089
Treatment Village	0.071	0.105	0.500	0.659	0.128	0.000	0.398	0.130	0.002	−1.337	0.131	0.000
Intercept	−1.461	0.265	0.000	−1.137	0.265	0.000	−2.374	0.306	0.000	1.385	0.269	0.000
												
Obs	2079.000			2079.000			2079.000			2079.000		
LR chi2(16)	242.100			324.990			173.430			163.530		
Pseudo R2	0.085			0.138			0.078			0.065		

Two further tests for endogenous exposure were also conducted. First, in the bivariate probit estimations (Table 3 and Table 7), an exogeneity test of ρ = 0, representing the correlation in the unobservables across the outcome and exposure equations, was conducted. We rejected the null of exogeneity for three out of four outcomes, the lone exception being for the outcome “agree that spacing is healthy.” In contrast, we failed to reject the null of exogeneity for any of the outcomes in the fixed effects, instrumental variables estimations, although in two cases p<0.10.

**Table 5:** Difference-in-Differences Estimations.

	Modern Contraceptive Use			Discussed FP with Spouse			Discussed Birth Spacing with Spouse			Agree that Birth Spacing is Healthy	
	Coef.	Std. Err.	P>z	Coef.	Std. Err.	P>z	Coef.	Std. Err.	P>z	Coef.	Std. Err.
Heard YHYW	0.0176	0.1380	0.898	−0.1206	0.1067	0.259	0.1606	0.1228	0.191	0.0055	0.1610
Year = 2005	0.1924	0.0594	0.001	−0.7601	0.1180	0.000	−0.5451	0.1243	0.000	−0.2990	0.1868
**YHYW x 2005**	**0.1238**	**0.1449**	**0.393**	**0.7708**	**0.1872**	**0.000**	**0.4961**	**0.1844**	**0.007**	**0.3943**	**0.2240**
Age (base = 15–19)											
20–24 years	0.6678	0.1578	0.000	0.0776	0.1555	0.618	−0.0950	0.1520	0.532	0.2705	0.1745
25–29 years	0.5486	0.1716	0.001	−0.1569	0.1644	0.340	−0.0968	0.1644	0.556	0.1884	0.1687
30–34 years	0.8983	0.1685	0.000	−0.6136	0.1895	0.001	−0.1190	0.1796	0.508	0.2066	0.1808
35–39 years	0.5795	0.1732	0.001	−1.3495	0.1870	0.000	−0.3875	0.1789	0.030	0.2165	0.1783
40–44 years	0.0922	0.1883	0.624	−2.1678	0.2489	0.000	−0.4970	0.2090	0.017	0.1431	0.2182
45–49 years	−1.3327	0.2454	0.000	−3.0716	0.3001	0.000	−0.5271	0.2236	0.018	−0.1228	0.2322
Children ever born	0.2613	0.0210	0.000	0.1474	0.0221	0.000	−0.0486	0.0171	0.005	0.0047	0.0194
Wealth (base = poorest)											
2nd poorest	0.0942	0.1137	0.407	0.1552	0.1145	0.175	0.4172	0.1243	0.001	−0.0631	0.1257
Middle	0.1486	0.1242	0.231	0.2358	0.1045	0.024	0.6356	0.1331	0.000	−0.0696	0.1275
2nd wealthiest	0.3588	0.1333	0.007	0.1635	0.1194	0.171	0.8751	0.1499	0.000	0.0098	0.1324
Wealthiest	0.4327	0.1293	0.001	0.3655	0.1165	0.002	0.9346	0.1358	0.000	0.3769	0.1555
Education (base = none)											
Primary	−0.0116	0.1038	0.911	−0.1471	0.1103	0.182	0.0566	0.1011	0.576	0.0575	0.1027
Secondary or above	−0.0774	0.2146	0.718	−0.2591	0.1855	0.163	−0.3913	0.2306	0.090	0.1083	0.1881
Has a community leader	0.1274	0.0857	0.137	−0.1010	0.0997	0.311	0.3680	0.0973	0.000	0.1856	0.1076
Lives in program village	0.0411	0.1245	0.741	0.3715	0.1042	0.000	0.3871	0.1057	0.000	−0.0793	0.1845
Intercept	−2.0565	0.2033	0.000	−0.6494	0.1556	0.000	−1.3700	0.2031	0.000	0.9053	0.2709
N	4163						4163			4163	
Wald chi2(18)	418.56			557.17			384.78			45.21	
Prob>chi2	0.00			0.00			0.00			0.00	

The measures of campaign effects derived by both the 2005 cross sectional estimates (Tables 3 and 4) and propensity score matching (PSM) (Table 3) were roughly similar. For example, the marginal effect of exposure to the “Your Health, Your Wealth” on modern contraceptive use was 3.9 percentage points by the 2005 cross sectional estimator, as compared with 4.0 percentage points for PSM, though in neither case were the results statistically significant. For the outcome “discuss birth spacing with spouse,” PSM and the 2005 cross sectional estimate were also similar –9.4 and 10.8 percentage points respectively.

**Table 6:** Fixed Effects Logit Results.

	Currently Using Modern Contraception			Discuss Family Planning with Spouse			Discussed Benefits of Birth Spacing			Agree that Birth Spacing improves health		
	Coef.	Std. Err.	P>z	Coef.	Std. Err.	P>z	Coef.	Std. Err.	P>z	Coef.	Std. Err.	P>z
**Heard YHYW**	0.3685	0.2811	0.190	0.9181	0.1936	0.000	0.6377	0.1887	0.001	0.2175	0.2248	0.333
Age (base = 15–19)												
20–29 years	−0.0044	0.4498	0.992	0.0902	0.4165	0.828	−0.1411	0.3653	0.699	0.1012	0.4060	0.803
30–39 years	0.4282	0.5936	0.471	−0.1709	0.5154	0.740	0.0632	0.4455	0.887	0.2428	0.5129	0.636
40–49 years	0.3232	0.7454	0.665	−0.5677	0.6420	0.377	−0.2104	0.5674	0.711	0.3819	0.5733	0.505
Children ever born	2.4096	0.2854	0.000	0.6994	0.1762	0.000	0.0163	0.1828	0.929	0.1616	0.2071	0.435
Wealth quintile (base = poorest)												
2nd poorest	−0.1735	0.3111	0.577	0.4629	0.2387	0.052	0.4928	0.2502	0.049	−0.0195	0.2166	0.928
Middle	−0.2591	0.3349	0.439	0.2501	0.2584	0.333	0.5157	0.2611	0.048	−0.3693	0.2366	0.119
2nd wealthiest	−0.0859	0.3564	0.809	0.2959	0.2685	0.270	0.8619	0.2738	0.002	−0.3006	0.2444	0.219
Wealthiest	−0.0396	0.4110	0.923	0.2966	0.3041	0.329	0.7271	0.3063	0.018	−0.4592	0.2957	0.120
Education (relative to none)												
Primary x Year = 2005	−0.1944	0.2993	0.516	0.0932	0.2299	0.685	0.3436	0.2073	0.097	−0.1370	0.2135	0.521
Secondary x Year = 2005	0.0760	0.5403	0.888	0.6393	0.3994	0.109	0.4207	0.4393	0.338	−0.5176	0.4675	0.268
Treatment Village x Year = 2005	−0.2858	0.2488	0.251	0.4576	0.1868	0.014	0.0436	0.1805	0.809	−2.2000	0.1781	0.000
Son should go to university	−0.0665	0.1574	0.673	−0.1483	0.1200	0.217	−0.4410	0.1184	0.000	−0.0869	0.1154	0.452
Year = 2005	−0.0794	0.2368	0.737	−1.4608	0.1829	0.000	−0.7922	0.1707	0.000	1.2191	0.1639	0.000
Obs	928			1512			1512			1710		
Groups	464			756			756			855		
LR chi2(14)	132.41			145.43			93.55			217.7		

The difference-in-differences models with control variables showed the effects of changes in family planning outcomes from 2004 to 2005 for exposed respondents relative to unexposed respondents. For all outcomes, the estimates were less than in the difference-in-differences models without controls. In two cases – discuss family planning with spouse and discuss birth spacing with spouse – exposure to the “Your Health, Your Wealth” messaging yielded positive marginal effects of 0.144 (p<0.001) and 0.086 (p = .021) respectively. For a third outcome (“Agree that spacing is healthy”), results were suggestive of a positive effect (marginal effect = 0.076; p = 0.068). For two of the four outcomes, the estimates were smaller than those estimated by PSM and the 2005 control function estimator.

**Table 7:** Bivariate Probit Estimates.

	Currently Using Modern Contraception			Discuss Family Planning with Spouse			Discussed Benefits of Birth Spacing			Agree that Birth Spacing improves health		
	Coef.	Std. Err.	P>z	Coef.	Std. Err.	P>z	Coef.	Std. Err.	P>z	Coef.	Std. Err.	P>z
**Heard YHYW**	0.6478	0.2158	0.003	1.381	0.1402	0.000	1.4268	0.1509	0.000	0.5739	0.2920	0.049
Age (base = 15–19)												
20–29 years	0.4196	0.0949	0.000	0.022	0.0836	0.795	−0.0528	0.0865	0.542	0.1625	0.0895	0.070
30–39 years	0.5767	0.1040	0.000	−0.425	0.0963	0.000	−0.1194	0.0973	0.220	0.1746	0.1012	0.084
40–49 years	−0.1600	0.1172	0.172	−1.247	0.1192	0.000	−0.2398	0.1094	0.028	0.0576	0.1142	0.614
Children ever born	0.1321	0.0131	0.000	0.078	0.0133	0.000	−0.0354	0.0132	0.007	0.0038	0.0141	0.787
CEB * year = 2005	0.0080	0.0152	0.600	−0.036	0.0160	0.023	0.0177	0.0159	0.267	−0.0075	0.0167	0.653
Wealth Quintile (Base = poorest)												
2nd poorest	0.0054	0.0665	0.936	0.038	0.0701	0.593	0.1769	0.0741	0.017	−0.0532	0.0709	0.453
Middle	0.0495	0.0675	0.464	0.074	0.0696	0.288	0.2917	0.0736	0.000	−0.0175	0.0713	0.807
2nd wealthiest	0.1679	0.0680	0.014	0.024	0.0708	0.737	0.4353	0.0735	0.000	0.0222	0.0724	0.760
Wealthiest	0.2027	0.0680	0.003	0.116	0.0704	0.100	0.4442	0.0731	0.000	0.2158	0.0759	0.004
Education (relative to none)												
Primary x Year = 2005	−0.0299	0.0555	0.591	−0.085	0.0596	0.154	0.0506	0.0590	0.391	0.0377	0.0592	0.524
Secondary x Year = 2005	−0.0260	0.1087	0.811	−0.139	0.1114	0.212	−0.2227	0.1102	0.043	0.0729	0.1164	0.531
Treatment Village x Year = 2005	0.0458	0.0629	0.466	0.093	0.0630	0.140	0.1997	0.0647	0.002	0.5723	0.0638	0.000
Treatment Village	−0.0149	0.0886	0.867	0.293	0.0937	0.002	0.0792	0.0936	0.397	−1.3114	0.1009	0.000
son_univ	−0.0264	0.0407	0.517	−0.123	0.0418	0.003	−0.2320	0.0421	0.000	−0.1137	0.0436	0.009
Year = 2005	−0.0272	0.1068	0.799	−0.687	0.1024	0.000	−0.6463	0.1022	0.000	0.6855	0.1476	0.000
Intercept	−1.1824	0.1156	0.000	−0.265	0.1083	0.014	−0.5368	0.1132	0.000	0.1960	0.1119	0.080
/atanhrho_12	−0.3532	0.1438	0.014	−0.7415	0.1299	0.0000	−0.7351	0.1403	0.000	−0.2381	0.1892	0.208
Obs	4165			4165			4165			4165		
Wald chi2(16)	393.99			570.34			411.9			255.25		
Prob > chi2	0.000						0.000			0.000		

The fixed effects logit estimator – incorporating individual-level fixed effects and using only time-varying characteristics of individuals - provided estimates of exposure that did not widely diverge from the simpler models. Again, exposure to the “Your Health, Your Wealth” campaign was shown to yield a 10.6 percentage point increase (p = 0.005) in the probability of discussing family planning with a spouse, lower than the 15.4 percentage point difference for PSM and 14.4 percentage point difference for the DID with controls. For the outcome “discussed spacing with spouse,” the marginal effect from the fixed effects model was 14.9 percentage points (p = 0.001), larger than the estimates of 9.4 percentage points from the PSM model and 8.6 percentage points from the DID model.

**Table 8:** Fixed Effects Instrumental Variables Estimates.

	Currently Using Modern Contraception			Discuss Family Planning with Spouse			Discussed Benefits of Birth Spacing			Agree that Birth Spacing improves health		
	Coef.	Std. Err.	P>z	Coef.	Std. Err.	P>z	Coef.	Std. Err.	P>z	Coef.	Std. Err.	P>z
**Heard YHYW**	0.2740	0.1355	0.043	0.6140	0.1991	0.002	0.2288	0.3005	0.446	0.2698	0.2662	0.311
Age (base = 15–19)												
20–29 years	0.0421	0.0528	0.425	−0.0092	0.0535	0.864	−0.0332	0.0663	0.616	0.0388	0.0657	0.555
30–39 years	0.0934	0.0761	0.220	−0.0378	0.0857	0.659	0.0006	0.0813	0.994	0.0328	0.0847	0.699
40–49 years	0.1025	0.0754	0.174	−0.0552	0.0939	0.557	−0.0397	0.0982	0.686	0.0892	0.1030	0.386
Children ever born	0.3518	0.0344	0.000	0.0633	0.0457	0.166	−0.0285	0.0399	0.474	−0.0062	0.0386	0.872
CEB * year = 2005	0.0166	0.0036	0.000	−0.0058	0.0048	0.224	0.0033	0.0064	0.600	−0.0031	0.0059	0.602
Wealth quintile (base = poorest)												
2nd poorest	−0.0230	0.0266	0.387	0.0524	0.0324	0.106	0.0685	0.0313	0.029	−0.0373	0.0348	0.283
Middle	−0.0256	0.0340	0.452	0.0145	0.0380	0.702	0.0597	0.0389	0.125	−0.1054	0.0485	0.030
2nd wealthiest	−0.0112	0.0326	0.731	0.0085	0.0423	0.842	0.1167	0.0482	0.016	−0.0992	0.0532	0.062
Wealthiest	−0.0278	0.0357	0.436	0.0094	0.0517	0.856	0.0894	0.0567	0.115	−0.0894	0.0592	0.131
Education (relative to none)												
Primary x Year = 2005	−0.0313	0.0272	0.250	0.0419	0.0350	0.231	0.0619	0.0393	0.115	0.0025	0.0358	0.945
Secondary x Year = 2005	−0.0013	0.0555	0.981	0.0804	0.0843	0.340	0.0735	0.0831	0.376	−0.1094	0.0644	0.089
Treatment Village x Year = 2005	0.0021	0.0231	0.929	0.0922	0.0391	0.018	−0.0018	0.0367	0.960	−0.3964	0.0673	0.000
Son should go to university	0.0027	0.0149	0.857	−0.0296	0.0185	0.109	−0.0801	0.0184	0.000	−0.0226	0.0262	0.389
Year = 2005	−0.13446	0.0439	0.002	−0.29787	0.0613	0.000	−0.15849	0.0839	0.059	0.186746	0.1006	0.063
Obs	4154			4154			4154			4154		
F(15,73)	11.85			7.25			6.53			4.83		
Centered R2	0.068			x			0.0368			0.0864		
Hansen J statistic (Overidentifcation test ofall instruments)	3.379			4.965			13.492			2.752		
Chi-sq(3) P-val =	0.3368			0.1744			0.0037			0.4315		
Endogeneity test	2.956			3.135			0.831			1.192		
Chi-sq(1) P-val =	0.0856			0.0766			0.3621			0.275		

Underidentification test (Kleibergen-Paap rk LM statistic = 33.647; Chi-sq(4) P-value = 0.000.

Weak identification test (Kleibergen-Paap rk Wald F statistic) = 16.551; Stock-Yogo weak ID test critical value (5% maximal IV relative bias = 16.85; 10% maximal IV relative bias = 10.27).

With the exception of the fixed effects logit models, the other methods controlling for possibly endogenous exposure tended to have considerably larger estimates of programmatic effects, providing support for the possibility that those most likely to be exposed to the “Your Health, Your Wealth” messages were those less likely to be influenced by the program. After controlling for both measured and unmeasured factors affecting non-random exposure, exposure to “Your Health, Your Wealth” was associated with an increase in modern contraceptive use by 23.1 percentage points (p = 0.002) in the bivariate probit model and by 27.4 percentage points (p = 0.043) in the fixed effects IV model. A similar pattern was evident for the outcome “discuss FP with one’s spouse” –42.5 percentage points (p<0.001) for the bivariate probit model and 61.4 percentage points (p = 0.002) for the fixed effects, IV estimator. Each of these estimates is many times larger than for the simpler models assuming exogenous exposure and even larger than the fixed effects logit estimator. The remaining two outcomes – “discussed birth spacing with spouse” and “agree that spacing is healthy for the baby” – were not statistically significantly related to exposure in the fixed effects IV models but they were for the bivariate probit models. In each case, the effects were several times larger than in the simpler models.

The considerable discrepancies between the simpler estimation methods and those addressing both the panel nature of the data and the possibility of endogenous exposure warrant careful examination. As noted by numerous researchers [49], [53], [54], instrumental variables estimators can yield widely misleading results if instruments are not carefully chosen. In order to be acceptable, these instrumental variables must be strongly associated with exposure but only minimally associated with our family planning outcomes (except indirectly through exposure). In this case, we have used several key variables that are hypothesized to affect exposure but not our family planning outcomes. These include self-reported watching of television every day, attendance at community gatherings, an index of other family planning campaigns that the respondent recalls, and a measure of local community social capital. By all appearances, these instrumental variables appear to meet the technical criteria for acceptability as instruments. The Kleibergen-Paap rk LM statistic of 33.666 (Table 8) allows us to reject the null hypothesis that our model is under-identified (p<0.001) [48]. We further reject the null hypothesis that we have weak instruments, as the estimated Kleibergen-Paap rk Wald F statistic is 16.668– slightly less than the Stock-Yogo critical value of 16.85 for 5% maximal IV relative bias but better than the critical value of 10.27 for 10% maximal IV relative bias [50]. As a result, there is some reason for confidence in the validity of our chosen instruments.

On the other hand, the fixed effects estimators rely on variation across time within individuals. In our data, only 378 out of 2,088 women reported being exposed to the “Your Health, Your Wealth” campaign over the time period. Further, within individual variation in many of the covariates appears minimal. Variables such as educational attainment could be included only by interacting with the time dummy. In light of these possible limitations, these results require some degree of caution.

## Discussion

This paper assesses the effects of exposure to a family planning health communication program – the “Your Health, Your Wealth” national multimedia campaign in Egypt – on a set of family planning outcomes, including current use of modern contraception, measures of interpersonal communication regarding family planning, and attitudes towards birth spacing. The aim of the paper is in large part methodological – to control appropriately for non-random (self-reported) exposure to the program in order to obtain more accurate measures of the program’s effects.

We make use of an atypically robust set of data – panel data with data collection occurring pre- and post-campaign and involving very low levels of attrition from the sample. The advantage of this data is that they allow for the use of estimation strategies that are not generally permitted by pooled cross-sectional data, a key limitation of many previous analyses of family planning communication efforts. Because the interventions are individualized – only through self-reported recall can researchers identify who is exposed and then trace that back to their pre-intervention outcomes and characteristics – cross-sectional models cannot identify changes across time in the treatment “exposed” group relative to the comparison “unexposed” group. Cross-sectional methods assuming exogenous exposure therefore assume that treatment and comparison individuals are statistically equivalent at baseline conditional on a limited set of control variables. Absent panel data, this assumption cannot be tested for time varying variables. Further, cross-sectional methods that attempt to control for endogenous exposure – instrumental variables methods or simultaneous equations models assuming a specific parametric distribution for the relationship between outcomes and exposure – must confront difficult issues of model identification or assume that identification is attained through assumptions about the parametric distribution.

By using panel data, in contrast, we can examine changes across time among a set of individuals who recall having been exposed to the campaign by the endline relative to individuals who do not recall such exposure. This allows for both difference-in-differences estimation and fixed effects estimation that can address changes across time or difference out unobserved heterogeneity affecting exposure and family planning outcomes. It also allows for the use of matching methods in which matching is determined by baseline characteristics of respondents rather than concurrently measured characteristics which may be more susceptible to underlying, unobserved heterogeneity.

The results, in this case, provide evidence that the “Your Health, Your Wealth” succeeded during the short time period of this study to achieve change in family planning outcomes. The models that made use of the panel nature of the data set – difference-in-differences, propensity score matching and fixed effects logit - provided similar results in terms of direction and levels of statistical significance but the magnitudes of effects often diverged widely. However, we find that estimates of program effects based on the perhaps naïve assumption that exposure is largely random once a limited set of observed covariates are controlled for may substantially underestimate program effects. In fact, we find that the magnitude of the underestimates could be on the order of three- to five-fold. For example, for two common methods assuming exogenous exposure – difference-in-differences models and propensity score matching – exposure to the “Your Health, Your Wealth” campaign was associated with an increase in modern contraceptive use of 2.9 and 4.0 percentage points respectively. In contrast, for the methods controlling for endogenous exposure – the bivariate probit and fixed effects IV estimator – the effect sizes were 23.1 and 27.4 percentage points respectively.

This is an important finding. If, for example, we applied the marginal effects from exposure to the “Your Health, Your Wealth” to a hypothetical population of 100,000 women exposed to the program, a doubling of the effect of the program – as was observed for the fixed effects IV model (ME = 0.270) relative to the difference-in-differences model (ME = 0.076) – would increase the number of women who agreed with the statement that “birth spacing is healthy” by approximately (27,000−7,600 = ) 19,400 women, an important programmatic result. Simpler methods such as the single equation cross-sectional estimator, which attribute to the program an increase in contraceptive use of 3.9 percentage points relative to the fixed effects IV estimate of only 27.4 percentage points, might similarly understate program effects.

In short, while the results from the different estimation methods are similar in direction and levels of statistical significance, the overall effects when applied at the population level can substantially alter conclusions about program success. Analysts and program managers who increasingly rely on estimates of program effects – particularly in estimates of cost-effectiveness - need to be cognizant of the limitations of their methods, particularly those based on cross sectional data.
